# Bromelain Inhibits Allergic Sensitization and Murine Asthma via Modulation of Dendritic Cells

**DOI:** 10.1155/2013/702196

**Published:** 2013-12-05

**Authors:** Eric R. Secor, Steven M. Szczepanek, Christine A. Castater, Alexander J. Adami, Adam P. Matson, Ektor T. Rafti, Linda Guernsey, Prabitha Natarajan, Jeffrey T. McNamara, Craig M. Schramm, Roger S. Thrall, Lawrence K. Silbart

**Affiliations:** ^1^Helen & Harry Gray Cancer Center and Department of Medicine, Hartford Hospital, 80 Seymour Street, P.O. Box 5037, Hartford, CT 06102-5037, USA; ^2^Department of Medicine, University of CT Health Center (UCHC), Farmington, CT 06030, USA; ^3^Department of Immunology, UCHC, Farmington, CT 06030, USA; ^4^Department of Pediatrics, UCHC, Farmington, CT 06030, USA; ^5^Department of Pathology, UCLA Immunogenetics Center, Los Angeles, CA 90095, USA; ^6^Department of Allied Health Sciences, University of CT, Storrs, CT 06268, USA

## Abstract

The incidence of atopic conditions has increased in industrialized countries. Persisting symptoms and concern for drug side-effects lead patients toward adjunctive treatments such as phytotherapy. Previously, we have shown that Bromelain (sBr), a mixture of cysteine proteases from pineapple, *Ananas comosus*, inhibits ovalbumin (OVA)-induced murine model of allergic airway disease (AAD). However, sBr's effect on development of AAD when treatment is administered throughout OVA-alum sensitization was unknown and is the aim of the present study. C57BL/6J mice were sensitized with OVA/alum and challenged with 7 days OVA aerosol. sBr 6 mg/kg/0.5 ml or PBS vehicle were administered throughout sensitization. Lung, bronchoalveolar lavage (BAL), spleen, and lymph nodes were processed for flow cytometry and OVA-specific IgE was determined via ELISA. sBr treatment throughout OVA-alum sensitization significantly reduced the development of AAD (BAL eosinophils and lymphocytes). OVA-specific IgE and OVA TET^+^ cells were decreased. sBr reduced CD11c^+^ dendritic cell subsets, and *in vitro* treatment of DCs significantly reduced CD44, a key receptor in both cell trafficking and activation. sBr was shown to reduce allergic sensitization and the generation of AAD upon antigen challenge. These results provide additional insight into sBr's anti-inflammatory and antiallergic properties and rationale for translation into the clinical arena.

## 1. Introduction

The incidence of atopic conditions such as asthma, food allergies and atopic dermatitis have increased dramatically in industrialized countries over the last fifty years. Presently, approximately 1 out of 5 Americans suffer from atopic disorders [1], with 1 out of 12 having asthma [2]. Despite major efforts to diagnose and treat these conditions, current conventional medications for allergic disorders are not fully effective. For example, it is estimated that 58% of primary care patients with asthma have poorly controlled asthma [3]. Poor asthma control may result from inadequate assessment or implementation of asthma therapy by healthcare providers or from poor adherence with prescribed therapy by patients [4]. The persistence of symptoms and disease flares, despite medical therapy and the concern for long-term side effects of corticosteroids [5–8] and long-acting beta-2 adrenergic agonists [9], have caused many patients to turn to complementary and alternative medicine (CAM) treatments [10]. A review of 17 articles reported that up to 70–80% of adult asthmatics in the USA use CAM to help control their asthma [11]. Similarly, the reported rates of CAM use in children with asthma range from 33 to 89% (11). In children, the most commonly used CAM therapies are breathing techniques, vitamins, and herbal products or phytotherapy [12].

One such herbal product with demonstrated anti-inflammatory efficacy is bromelain. Stem bromelain (sBr) is a mixture of cysteine proteases that is derived from the stem of the pineapple plant, *Ananas comosus*. Beneficial effects of sBr have been demonstrated in a variety of inflammatory conditions, including rheumatologic diseases in mice and humans [8–13], experimental allergic encephalomyelitis (a murine model of multiple sclerosis) [14], human allergic rhinitis [15], and murine allergic asthma [16]. In an ovalbumin- (OVA-) induced asthma model [17], we have shown that sBr administered by either intraperitoneal [16] or oral routes [18] inhibits eosinophilic airway inflammation and allergic airway disease, at least in part via proteolytic cleavage of cell-surface CD25 from activated CD4^+^ T effector cells [19]. In these experiments, sBr was administered prior to and during OVA aerosol challenges (but following OVA antigen sensitization). It is noted that sBr can also affect many other cell surface markers common to T cells and other cell types such as dendritic cells (DC) which could potentially affect their function.

Dendritic cells are professional antigen-presenting cells and are known to take up antigen via specialized endocytic receptors and in response to danger signals and migrate to sites of inflammation [20–22]. Although identifying the specific subsets of DCs which migrate and are responsible for antigen uptake and presentation remains an active area of research, CD44, the receptor for hyaluronic acid, has been shown to be essential for DC migration to regional lymph nodes [23], Th2 skewed T-cell activation [24, 25], and inflammation [26]. Interestingly, sBr has been shown to reduce the expression of CD44 in a variety of models of cell adhesion [27–29] and metastasis [30, 31], thus presenting a plausible mechanism for inhibition of allergic sensitization. Thus, the present study addressed the hypothesis that sBr could inhibit murine sensitization to OVA via modulation of DCs, which play a key role in allergic sensitization.

## 2. Methods

### 2.1. Animals

Female C57BL/6J mice, 3–6 months of age, Jackson Laboratory (Bar Harbor, ME), were housed in plastic cages with corncob bedding at 22–24°C with a daily light/dark cycle (light from 06:00 to 18:00 h). Chow and water were supplied *ad libitum*. All protocols were approved by the UConn Animal Care Committee.

### 2.2. Natural Product Bromelain

For intraperitoneal (i.p.) injections, 60 mg sBr (Vital Nutrients, Middletown, CT) was dissolved in 250 ml PBS. sBr was independently tested for authenticity, potency (2400–2660 GDU g^−1^), and quality as previously described [16, 18, 19].

### 2.3. *In Vitro* Bromelain Studies

sBr was administered in a dose response manner (1–100 *μ*g/mL) to DCs overnight. To obtain DCs, spleens of OVA-alum sensitized mice were digested with Collagenase-D (Roche, Indianapolis, IN) 2 mg/mL for 30 m at 37°C, passed through a 40 *μ*m nylon cell strainer (BD, Bedford, MA) and erythrocytes lysed with Tris-buffered ammonium chloride at room temperature for ~2 min. CD11c^+^ cells were then isolated with pan-CD11c microbeads (number 130-092-465; Miltenyi Biotech, Auburn, CA). CD11c^+^ cell isolations yielded 3–5 × 10^6^ cells/spleen with a purity of >95%. Cells (0.5–1 × 10^6^) were cultured in 24 well plates in CO_2_ incubator (5%, 37°C). 100 *μ*M E-64 (Sigma, St. Louis, MO) was added to neutralize sBr cysteine protease activity, in selected experiments.

### 2.4. Bromelain Treatment in OVA-Induced Murine Models of Allergic Airway Disease and OVA/Alum Sensitization

**Figure 1:**
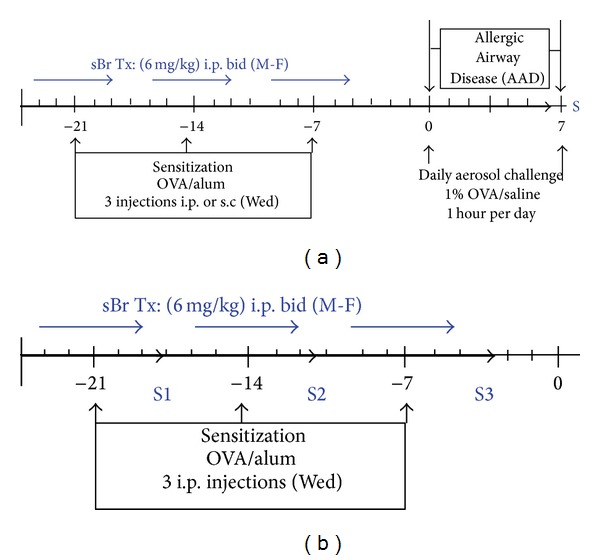
Protocols for sBr treatment in murine models of allergic airway disease. (a) Mice (*n* = 8–10 per group) were sensitized to OVA-alum (i.p.) weekly, for 3 consecutive weeks. sBr (6 mg/kg in 0.5 ml PBS) or PBS was delivered i.p. twice daily. On day 0, mice were challenged with OVA aerosol for seven consecutive days and sacrificed (S) 24 hours later. (b) Sensitization and i.p. sBr treatment were the same as in (a). Animals were sacrificed 24 hrs after each week or treatment; S1 (week 1), S2 (week 2), and S3 (week 3). In selected experiments groups of mice were treated with E64-inhibited-sBr.

Mice were sensitized with three weekly i.p. or subcutaneous (nape of neck) injections of a suspension containing 25 *μ*g of OVA (grade V, Sigma Chemical, St. Louis, MO) and 2 mg of aluminum hydroxide (alum) in 0.5 mL of saline (Figure 1(a)). OVA-alum was delivered i.p. once per week for 3 consecutive weeks (days −21, −14 and −7) to C57BL/6J mice (Figure 1(b)) PBS or sBr was delivered i.p. (6 mg/kg in 0.5 ml PBS) twice daily, M-F throughout sensitization. In prior studies we determined that sBr (6 mg/kg in PBS) administered i.p. twice daily for 3 consecutive weeks caused no significant elevation in liver enzymes or BAL protein in these animals (see Supplemental Table 1 in Supplementary Materials available at http://dx.doi.org/10.1155/2013/702196).

After sensitization, animals were rested for 1 week and then challenged with 1% aerosolized OVA in 0.9% saline, 1 h per day, for seven days (days 0–7), [16, 19]. Twenty-four hours after the final aerosol exposure, the mice were sacrificed by drug overdose (0.15 mL i.p. injection per 20 g mouse of 13 mg Ketamine HCL, Ketaset-III For Dodge Animal Health, Fort Dodge, IA, USA, and 0.4 mg of xylazine, Tranquived Vedco, St. Joseph, MO, USA) and exsanguination.

Animals were also sacrificed 24 hours after each weekly sensitization (Figure 1(b)); S1 (week 1), S2 (week 2), and S3 (week 3), and tissues (spleen, lymph nodes, and BAL) were processed for assessment of the antigen-specific responses. In selected experiments, a group of mice treated with E64-inhibited-sBr was added as a control for the cysteine protease activity of sBr. The sBr dosages used were based on prior *in vivo* and *in vitro* dose response studies performed in our laboratory [16, 18, 19].

### 2.5. BAL Cellular Analysis

Lungs were lavaged *in situ *with five 1 mL aliquots of 0.9% saline. BAL fluid was centrifuged (200 g × 10 min), the pellet was resuspended in saline, and total nucleated cells were counted with a hemocytometer using Nigrosin exclusion for viability. Leukocyte differentials were determined using cytocentrifuged (at 900 rpm for 5 min, Thermo Scientific Shandon Cytospin-4, Leicestershire, England, UK) preparations stained with May-Grünwald and Giemsa (Accustain, Sigma, St Louis, MO, USA). The remaining cells were analyzed phenotypically for T-cell subpopulations by flow cytometry.

### 2.6. Flow Cytometry

Cells for analysis via fluorescence-activated cell sorting (FACS) were obtained from BAL, homogenized lung tissue, spleen, and lymph nodes. BAL samples were washed in PBS (Dulbecco's Phosphate Buffered Saline, pH 7.4, Sigma, St Louis, MO, USA) and tissues processed and labeled with monoclonal antibodies in standard manner for flow cytometry. The following monoclonal antibodies were used for cellular surface staining: *α*-CD3 (145-2c11), CD4 (RM4-5), CD8 (53-6.72), *α*-CD11a (2D7), CD44 (IM7), *α*-CD62L (MEL-14), CD86 (GL-1), CD11b (M1/70), CD103 (2E7), CD11c (N418), F4/80 (BM8), and MHCII (M5/114.15.2) and were purchased from eBioscience (San Diego, CA) or BioLegend (San Diego, CA). H-2K^b^ tetramers containing the OVA-derived peptide SIINFEKL were generated in the laboratory as described previously [32]. Enrichment of OVA-TET^+^ CD8^+^ T cells from mice was accomplished by processing single cell suspensions from the spleen or pooled lymph nodes (axillary, mandibular, cervical, HLN, ILN, colic, jejunal, and caudal mesenteric). Cells were then stained with both phosphatidylethanolamine- and allophycocyanin (APC)-labeled tetramers and *α*-CD8 antibody then counter-stained with *α*-phosphatidylethanolamine microbeads as per the instructions of the manufacturer (Miltenyi Biotec, Auburn, CA). Samples were then run on an AutoMACs (Miltenyi Biotec) magnetic column cell separator. After enrichment, cells were stained with *α*-CD11a, *α*-CD62L, *α*-CD4, *α*-IA^b^, and *α*-CD11b for 30 minutes at 4°C. Cells were then washed and fixed with 2% paraformaldehyde. Cell samples were acquired with an LSRII cytometer (Becton Dickinson Biosciences, San Jose, CA) and analyzed with FlowJo software (Tree Star, Inc., Ashland, OR). General gating strategies are depicted in Supplementary Figure 3.

### 2.7. Statistical Analysis

Statistical comparisons between groups were made with analysis of variance and unpaired *t*-tests using JMP Software (SAS Institute Inc., Cary, NC, USA). All data were expressed as means ± standard error of the mean, and differences were considered significant at *P* ≤ 0.05.

## 3. Results

### 3.1. Bromelain Administration during Sensitization Prevented Development of Allergic Airway Disease

**Figure 2:**
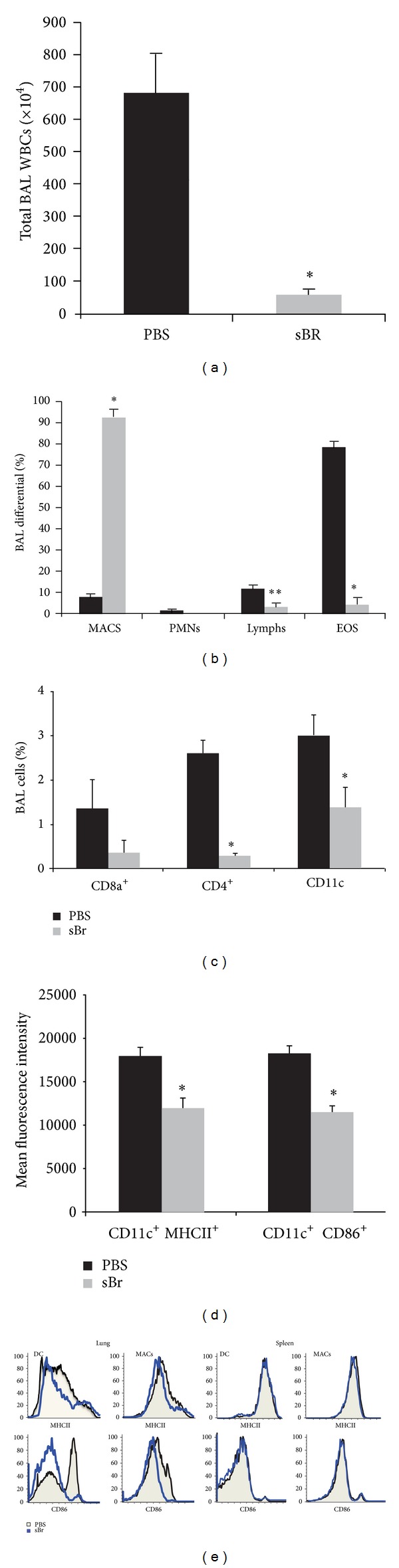
Bromelain treatment through sensitization abrogates the development of AAD upon OVA aerosol challenge. As compared to PBS treated controls, sBr treatment significantly reduced total BAL WBCs (a) as well as lymphocytes and eosinophils (b). sBr also inhibited influx of BAL CD4^+^ and CD8^+^ T lymphocytes and % of CD11c^+^ cells and mean fluorescent intensity of CD86 and MHCII (c). A representative FACS plot (e) compares the MHCII and CD86 expression on lung DCs (CD11c^+^F480^−^) and MACs (CD11c^+^F480^+^) with those in the spleen (*n* = 8–10 per group).

In the current studies, treatment of mice with bromelain during the sensitization prevented the development of AAD in the animals (Figure 2). Total BAL leukocytes were markedly reduced in sBr-treated AAD mice as compared to control AAD animals (PBS controls 683.8 ± 120 × 10^4^; sBr 58.1 ± 16.7 × 10^4^ cells; *P* < 0.0001; Figure 2(a)). In regards to the BAL WBC differentials (Figure 2(b)), MACs remained prevalent with sBr treatment (PBS treated 8.0 ± 1%; sBr 92 ± 4%); *P* < 0.0001), lymphs (PBS treated 11.9 ± 1.7%; sBr 3.3 ± 1.8%; *P* < 0.0001), and EOS (PBS treated 78.5 ± 2.8%; sBr 4.3 ± 3%; *P* < 0.0001) were significantly reduced and PMNs (PBS treated 1.6 ± 1%; sBr 0 ± 0%) remained unchanged.

Similar effects were observed in the OVA-alum subcutaneously sensitized mice, with i.p administration of sBr (Supplementary Figure 4). sBr decreased total BAL leukocytes (PSB control 64 ± 8 × 10^5^, sBr 18 ± 4 × 10^5^; *P* < 0.001) as well as BAL eosinophils (PSB control 56 ± 8 × 10^5^, sBr 14 ± 3 × 10^5^; *P* < 0.001), *n* = 8 animals per group. These observations confirmed that the inhibitory effect of i.p. sBr was not due to allosteric interaction with the i.p. OVA.

### 3.2. Bromelain Administration during Sensitization Reduced Regional Lymphocytes and Cell Activation after OVA Aerosol Challenge

The above BAL differentials showed that the inhibition of allergic airway disease by sBr treatment was accompanied by an absence of airway lymphocytosis in treated animals. Subsequent FACS analysis demonstrated marked reductions in BAL CD4^+^ and CD8^+^ T cells as well as CD11c^+^ cells in sBr-treated animals (Figure 2(c)). Accompanying the regional reduction in T-cell numbers, the mean fluorescence intensity (MFI) of activation markers MHCII and CD86 were significantly reduced on CD11c^+^ DCs in lung tissue of sBr-treated mice (Figure 2(d)). A representative histogram (Figure 2(e)) demonstrates that this reduction in receptor expression was predominant in CD11c^+^F4/80^−^ DCs localized to the lung tissue as compared to the spleen.

### 3.3. Bromelain Administration during Sensitization Inhibited Antigen-Specific Immunoglobulin Production

**Figure 3:**
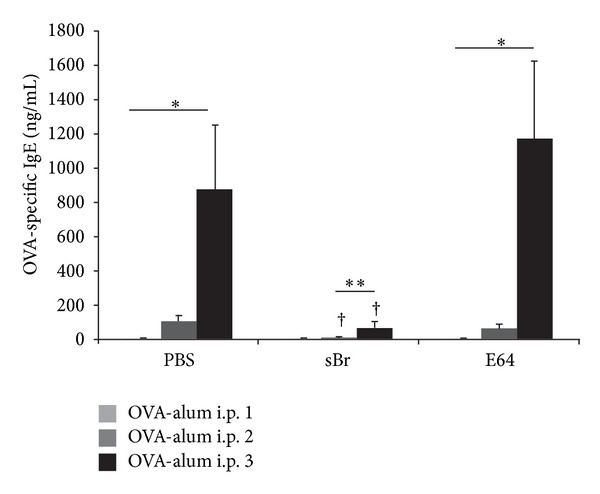
sBr treatment throughout sensitization reduces production of antigen-specific IgE. Peripheral blood of mice (PBS, sBr, or E64-inhibited-sBr) was collected after each weekly sensitization and serum was processed for concentration of OVA-specific IgE. In PBS-treated controls, a significant increase in the concentration of OVA-specific IgE was noted after i.p.'s 2 and 3 when compared to i.p. 1. In sBr-treated groups, the production OVA-specific IgE was delayed and attenuated relative to PBS groups. IgE production was restored in the E64 treatment group. PBS: phosphate buffered saline, E64: E64-inhibited-sBr (*P < 0.001, **P < 0.05 between i.p.'s; ^†^ *P* < 0.01 between PBS treated groups; *n* = 4 per group).

The prevention of AAD in mice treated with bromelain during OVA sensitization suggested that bromelain interfered with the sensitization process. This consideration was first addressed by measurement of OVA-specific IgE levels after each of the OVA-alum injections. As expected, i.p. sensitization with OVA-alum resulted in the production of OVA-specific IgE. Serum OVA-specific IgE levels increased from non-detectable levels in naïve animals to 869 ± 379 ng/mL following the third i.p. injection (Figure 3). This increase was markedly inhibited in sBr-treated animals, with a final IgE level of only 71 ± 37 ng/mL. The attenuation was due to the proteolytic action of sBr, since treatment of sBr with the antiprotease E64 abolished the effect. Mice treated with E64-treated sBr developed OVA-specific IgE levels of 1165 ± 461 ng/mL after the third i.p sensitization.

### 3.4. Bromelain Treatment throughout Sensitization Prevented Generation of an OVA Specific CD8^+^ T-Cell Response

**Figure 4:**
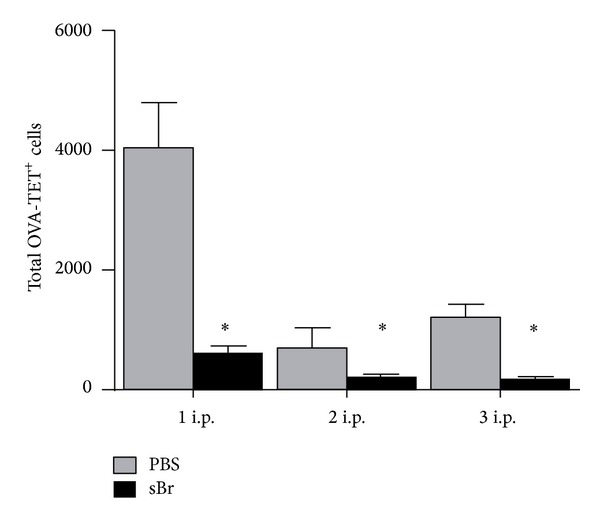
sBr treatment throughout sensitization prevents generation of OVA-specific CD8^+^ T-cell response. Animals were sacrificed after each weekly OVA-alum i.p. Spleen and nodes were pooled and enriched for OVA-specific CD8^+^ T cells via SIINFEKL (OVA_257–264_) loaded tetramer. As compared to PBS-treated controls, sBr treatment significantly limited the expansion of total OVA-TET^+^ CD8^+^ T cells after each weekly OVA-alum i.p. (*P < 0.001 compared to PBS; *n* = 3–5 per group).

As in Figure 1(b) Protocol-, PBS-, or sBr-treated animals were sacrificed after each weekly OVA-alum i.p. Spleen and nodes (mediastinal, cervical, axillary, brachial, inguinal, and mesenteric) were pooled and enriched for OVA-specific CD8^+^ T cells via SIINFEKL (OVA_257–264_) loaded tetramer. As compared to PBS-treated controls, sBr treatment significantly limited the expansion of total OVA-TET^+^ CD8^+^ T cells after each weekly OVA-alum i.p. (Figure 4).

### 3.5. Bromelain Administration during OVA-Alum Sensitization Reduced DCs in the MLNs

**Figure 5:**
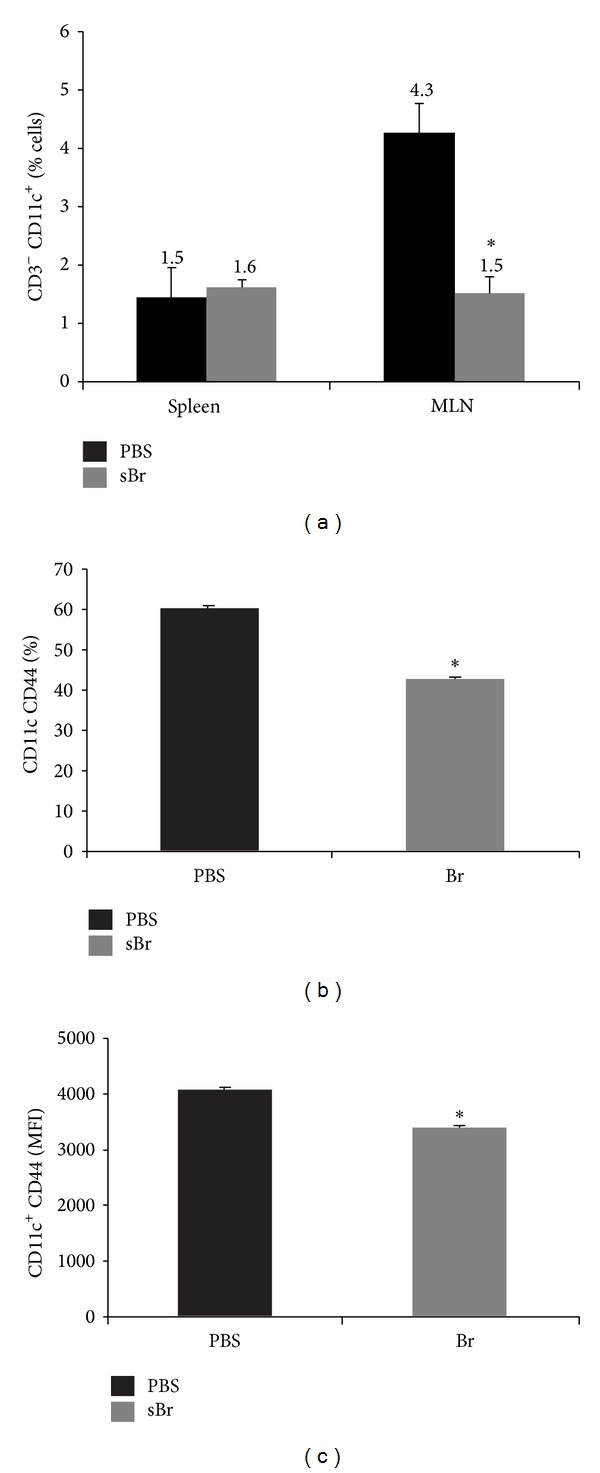
sBr treatment through OVA/alum sensitization reduces DCs in the MLN. Animals were treated with sBr and PBS through the first OVA-alum i.p. Upon sacrifice spleen, and pooled MLN (mesenteric lymph nodes) were processed for analysis via flow cytometry. CD3^−^ CD11c^+^ DCs were reduced in MLNs in sBr-treated animals as compared to PBS-treated controls. Both the percentage of CD44^+^ cells (b) and the mean fluorescent intensity (MFI) of CD44 (c) were significantly reduced in the sBr treatment group as compared to the control PBS group. *P < 0.05; *n* = 5 animals per group.

The attenuation of both IgE and T-cell responses to OVA demonstrated that sBr modulated pathways involved in allergic sensitization. One key pathway involves antigen presentation by DCs; therefore, we wanted to determine if sBr altered DCs after sensitization. As in Figure 1(b), sBr was administered throughout the 1st OVA-alum i.p., and DCs subtypes were evaluated in the spleen and MLNs. sBr did not affect the number of splenic DCs in OVA-sensitized mice, as assessed by percentage of CD11c^+^ cells. However, the percentage of DCs in the MLNs was significantly reduced (Figure 5(a)) by sBr administration (PBS 4.3 ± 0.5%, sBr 1.5 ± 0.2%; *P* < 0.0001). Both the percentage (Figure 5(b)) and MFI of CD44 were significantly reduced *in vivo*. In addition, the total CD11c^+^ cells were reduced in the MLN in CD103 (Supplementary Figure  4(a)), CDllb^+^CD8^+^ (Supplementary Figure  4(b)) and CDllb^−^CD8^+^ (Supplementary Figure  4(c)) subsets.

### 3.6. *In Vitro* sBr Treatment of DCs Reduced CD44

**Figure 6:**
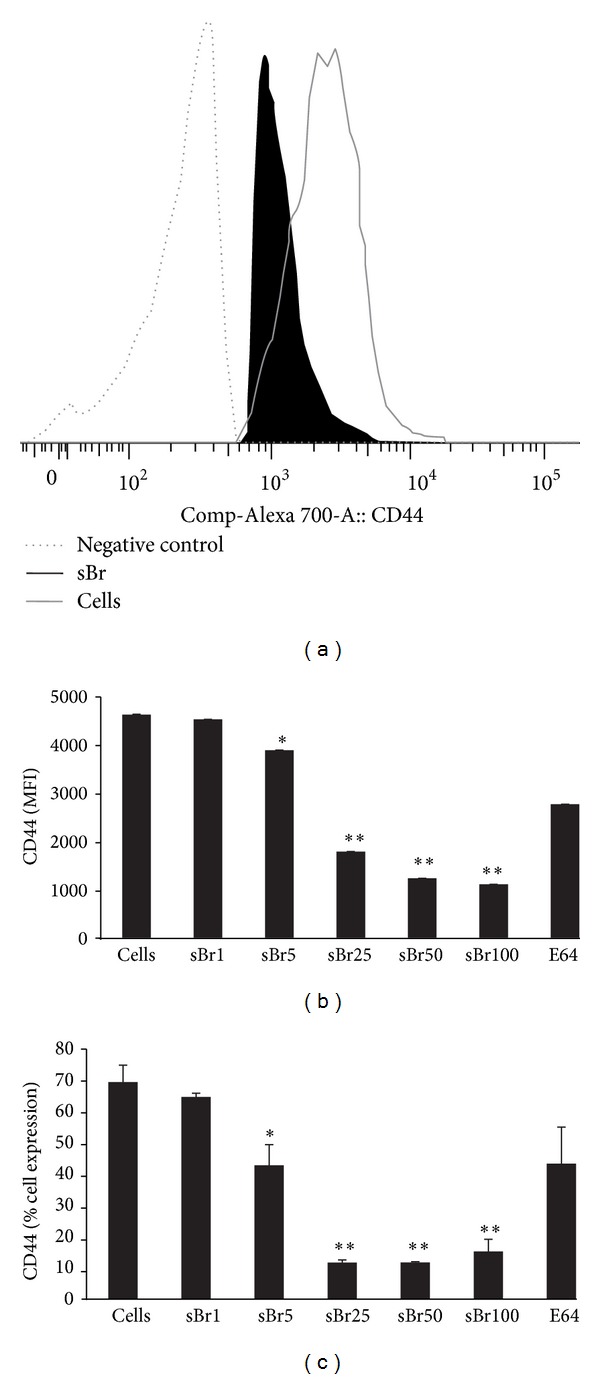
sBr treatment of DCs *in vitro* results in reduced expression of CD44. MLN DCs were selected using Pan DC Beads and cells (5 × 10^5^/well) were cultured overnight. (a) Representative histogram of CD44 expression on cells in media alone (MFI 2668), sBr 100 *μ*g/mL treated cells MFI (1198), and negative control (MFI 244). (b) denotes MFI of CD44 and (c) % cell expression of CD44 on cells alone, sBr (1−100 *μ*g/mL), and (E64 treated sBr 100 *μ*g/mL). Gates were on live, CD3^−^CD11c^+^MHCII^+^ cells; MFI: mean fluorescence intensity. *P < 0.01; **P < 0.0001 as compared to control cells in media alone. Data represents triplicate wells of duplicate experiments.

Noting that there was a reduction in DCs in the MLNs in addition to CD44 expression with *in vivo* sBr treatment, we wanted to confirm *in vitro* that sBr was having specific effect on DC receptor expression. Therefore, CD11c^+^ DCs were isolated via positive selection with pan CD11c microbeads and cultured overnight with escalating doses of sBr (1–100 *μ*g/mL) and 100 *μ*M E-64 inhibited sBr 100 *μ*g/mL (Figure 6). As compared to cells in media alone, sBr treatment did not alter the expression of CD11c or MHCII (data not shown). However, CD44 expression was reduced by sBr at doses of 5 *μ*g/mL and greater (Figure 6(b)), with similar reductions in CD44 noted between the 5 and 100 *μ*g/mL doses (Figure 6(c)).

## 4. Discussion

In previous studies, we demonstrated that i.p. or oral administration of sBr before, during, or after antigen challenge blunts the development of AAD in previously OVA-sensitized mice [16, 18, 19]. The present study extended those findings to demonstrate that sBr given during the sensitization markedly diminished the AAD response to subsequent aerosolized OVA challenge (~90%), despite the absence of sBr treatment during the aerosol challenge period. The BAL of naïve mice and mice undergoing OVA sensitization but no OVA-aerosol challenge consists of >95% macrophages with minimal presence of other cell types [19]. This differential distribution of BAL cell types was not affected during the 3 weeks of sensitization by concomitant administration of sBr (Supplementary Figure 1). In contrast, allergic airway disease (AAD), induced by 3–10 days of aerosolized OVA challenges to sensitized mice, is characterized by marked elevations in eosinophils and lymphocytes [19]. sBr exerted profound inhibitory effects on OVA sensitization itself, resulting in marked reductions in serum OVA-specific IgE and generation of OVA-TET^+^ CD8^+^ T cells. The lack of allergic sensitization was accompanied by a reduction in DCs in the MLN, the percentage of CD44^+^ cells, and a reduction in expression of CD44, a key modulator of DC activation and migration, *in vitro*.

CD44 is one of the most sensitive surface markers to bromelain degradation. The ~80% reduction in CD44 expression with sBr treatment noted in these *in vitro* studies is similar to the >90% reduction reported by Hale and colleagues in CD44 levels in human peripheral blood lymphocytes incubated for 1 hour in the presence of sBr [27]. CD44 is involved in a number of important biological processes including lymphocyte activation and homing, hematopoiesis, and tumor progression, and metastasis [30]. Of particular relevance to allergies and asthma, it has been shown that CD44 expression on DCs plays a crucial role in DC activation of T cells. The engagement of CD44 molecules expressed on the surface of DCs by specific mAbs or by its extracelluar matrix ligand, hyaluronic acid, induces DC phenotypic and functional maturation [31]. This maturation is associated with increased expression of several surface markers, including HLA class II molecules, and increased allogeneic T cell stimulatory capacity [33–35]. CD44 receptor activity is induced by antigen stimulation in antigen-sensitized spleen CD4^+^ T cells, and T-cell expression of CD44 is important for the accumulation of antigen-specific Th2 cells in the airway and in the development of AAD induced by antigen challenge [32, 36]. Thus, sBr-induced reduction of CD44 on DCs could have resulted in impaired sensitization to antigen. Future studies will investigate the role of sBr on antigen uptake and presentation in DCs to determine the degree to which DC function is altered.

In summary, the present study demonstrated that sBr attenuated the AAD response when administered throughout sensitization. Additionally, sBr prevented allergic sensitization, which was attributed to reduced accumulation of DCs in the MLNs and decreased CD44 expression of treated DCs *in vitro.* Future research may confirm sBr's role and the specific components within sBr [37] which modulate antigen uptake and presentation in DCs. Both pathways are likely targets in combating allergies. These findings identified an additional inhibitory mechanism of sBr on allergic responses and further support the potential utility of this CAM product in the treatment of allergies and asthma.

## Supplementary Material

In supplementary Table 1 toxicity parameters (liver function, bronchoalveolar lavage (BAL) protein analysis and total WBCs) determine that sBr treatment was not harmful over the three week treatment course. Supplementary Figure 1 determines that sBr treatment, throughout OVA/alum sensitization, did not alter the BAL cellular differential. In order to determine if the co-localization of i.p. sBr treatment and i.p OVA/alum sensitization resulted in the reduced allergic airway disease (AAD) the immunization and sBr treatment were separated. Supplementary Figure 2 provides data showing that sBr i.p. treatment still reduced BAL leukocytes at AAD, with subcutaneous OVA/alum sensitization. Supplementary Figure 3 depicts the general gating strategies for flow cytometry and Supplementary Figure 4 illustrates the reduction of DC subsets (in the mesenteric lymph nodes) when sBr is administered throughout OVA/Alum sensitization.
