# Supplementary material for: Bromelain Inhibits Allergic Sensitization and Murine Asthma via Modulation of Dendritic Cells
Source: Evid Based Complement Alternat Med. 2013 Dec 5;2013:702196. doi: 10.1155/2013/702196 (PMC3870104; doi:10.1155/2013/702196)
Supplement: Supplementary file 1 — In supplementary Table 1 toxicity parameters (liver function, bronchoalveolar lavage (BAL) protein analysis and total WBCs) determine that sBr treatment was not harmful over the three week treatment course. Supplementary Figure 1 determines that sBr treatment, throughout OVA/alum sensitization, did not alter the BAL cellular differential. In order to determine if the co-localization of i.p. sBr treatment and i.p OVA/alum sensitization resulted in the reduced allergic airway disease (AAD) the immunization and sBr treatment were separated. Supplementary Figure 2 provides data showing that sBr i.p. treatment still reduced BAL leukocytes at AAD, with subcutaneous OVA/alum sensitization. Supplementary Figure 3 depicts the general gating strategies for flow cytometry and Supplementary Figure 4 illustrates the reduction of DC subsets (in the mesenteric lymph nodes) when sBr is administered throughout OVA/Alum sensitization. [file 702196.f1.zip › TabS1.pdf]

Supplemental Table 1

	PBS	sBr	Sig
**Liver Function**			
**AST**	41.2 ± 14	41.8 ± 12	*P* = 0.94
**ALP**	83.6 ± 11	60.4 ± 12	*P* = 0.05
**Total Protein**	4.77 ± 0.2	3.42 ± 0.2	*P* = 0.15
**Albumin**	5.40 ± 1.5	5.20 ± 0.7	*P* = 0.21
**Lung Injury**			
**BAL BCA (pg/ml)**	166 ± 27	129 ± 10	*P* = 0.05
**BAL Total WBC (x 10^4)**	4.7 ± 4.0	5.6 ± 3.4	*P* = 0.19
