# Supplementary figures and images for: Bromelain Inhibits Allergic Sensitization and Murine Asthma via Modulation of Dendritic Cells
Source: Evid Based Complement Alternat Med. 2013 Dec 5;2013:702196. doi: 10.1155/2013/702196 (PMC3870104; doi:10.1155/2013/702196)

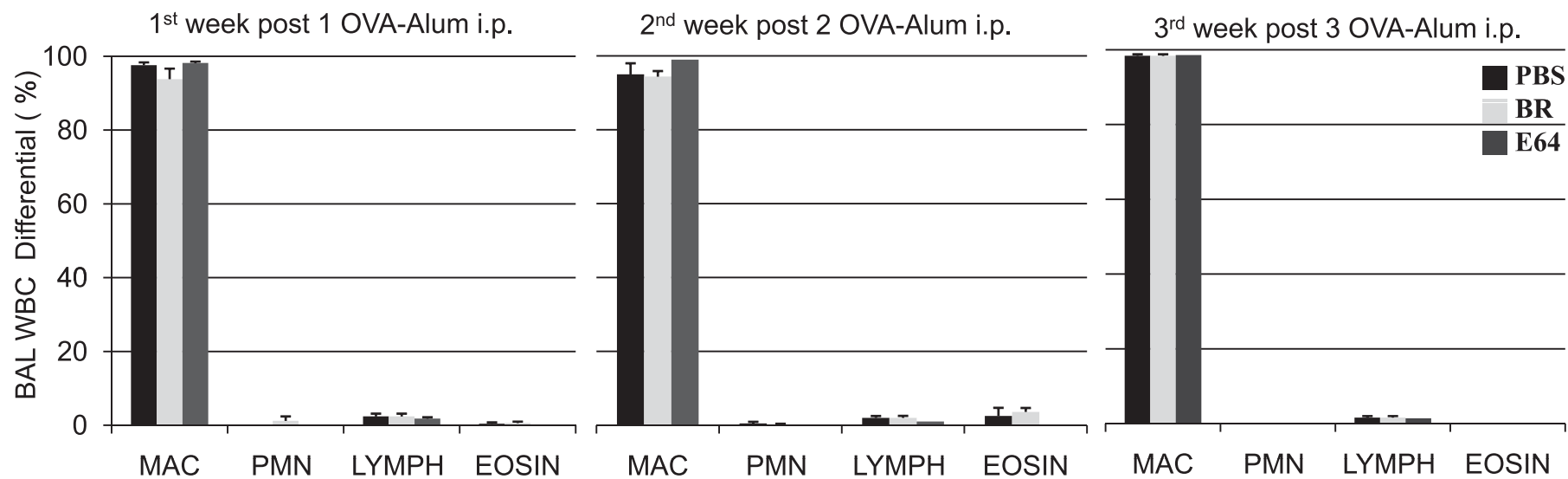

Supplement: Supplementary file 1 — In supplementary Table 1 toxicity parameters (liver function, bronchoalveolar lavage (BAL) protein analysis and total WBCs) determine that sBr treatment was not harmful over the three week treatment course. Supplementary Figure 1 determines that sBr treatment, throughout OVA/alum sensitization, did not alter the BAL cellular differential. In order to determine if the co-localization of i.p. sBr treatment and i.p OVA/alum sensitization resulted in the reduced allergic airway disease (AAD) the immunization and sBr treatment were separated. Supplementary Figure 2 provides data showing that sBr i.p. treatment still reduced BAL leukocytes at AAD, with subcutaneous OVA/alum sensitization. Supplementary Figure 3 depicts the general gating strategies for flow cytometry and Supplementary Figure 4 illustrates the reduction of DC subsets (in the mesenteric lymph nodes) when sBr is administered throughout OVA/Alum sensitization. [file 702196.f1.zip › FigS1.pdf]

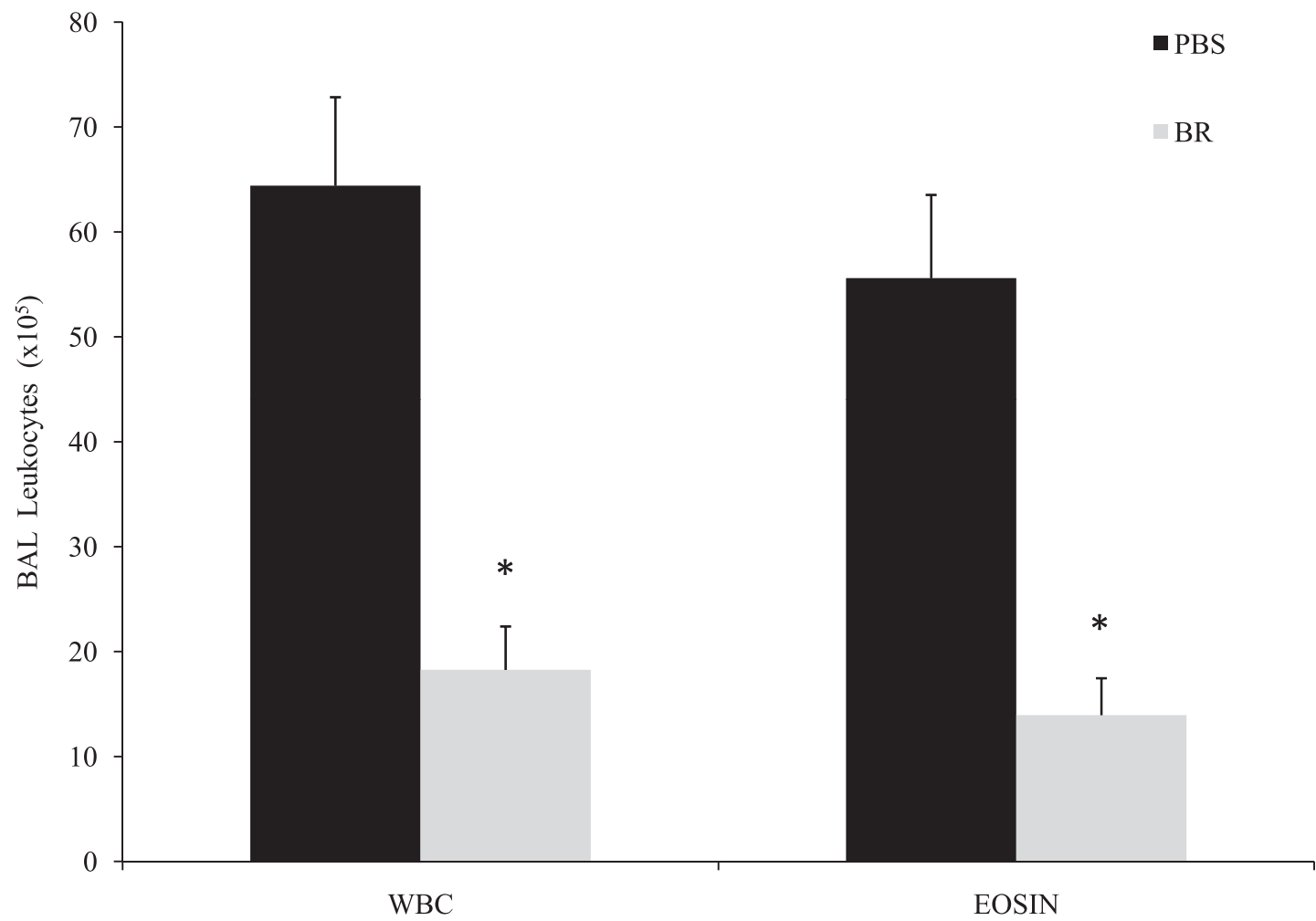

Supplement: Supplementary file 1 — In supplementary Table 1 toxicity parameters (liver function, bronchoalveolar lavage (BAL) protein analysis and total WBCs) determine that sBr treatment was not harmful over the three week treatment course. Supplementary Figure 1 determines that sBr treatment, throughout OVA/alum sensitization, did not alter the BAL cellular differential. In order to determine if the co-localization of i.p. sBr treatment and i.p OVA/alum sensitization resulted in the reduced allergic airway disease (AAD) the immunization and sBr treatment were separated. Supplementary Figure 2 provides data showing that sBr i.p. treatment still reduced BAL leukocytes at AAD, with subcutaneous OVA/alum sensitization. Supplementary Figure 3 depicts the general gating strategies for flow cytometry and Supplementary Figure 4 illustrates the reduction of DC subsets (in the mesenteric lymph nodes) when sBr is administered throughout OVA/Alum sensitization. [file 702196.f1.zip › FigS2.pdf]

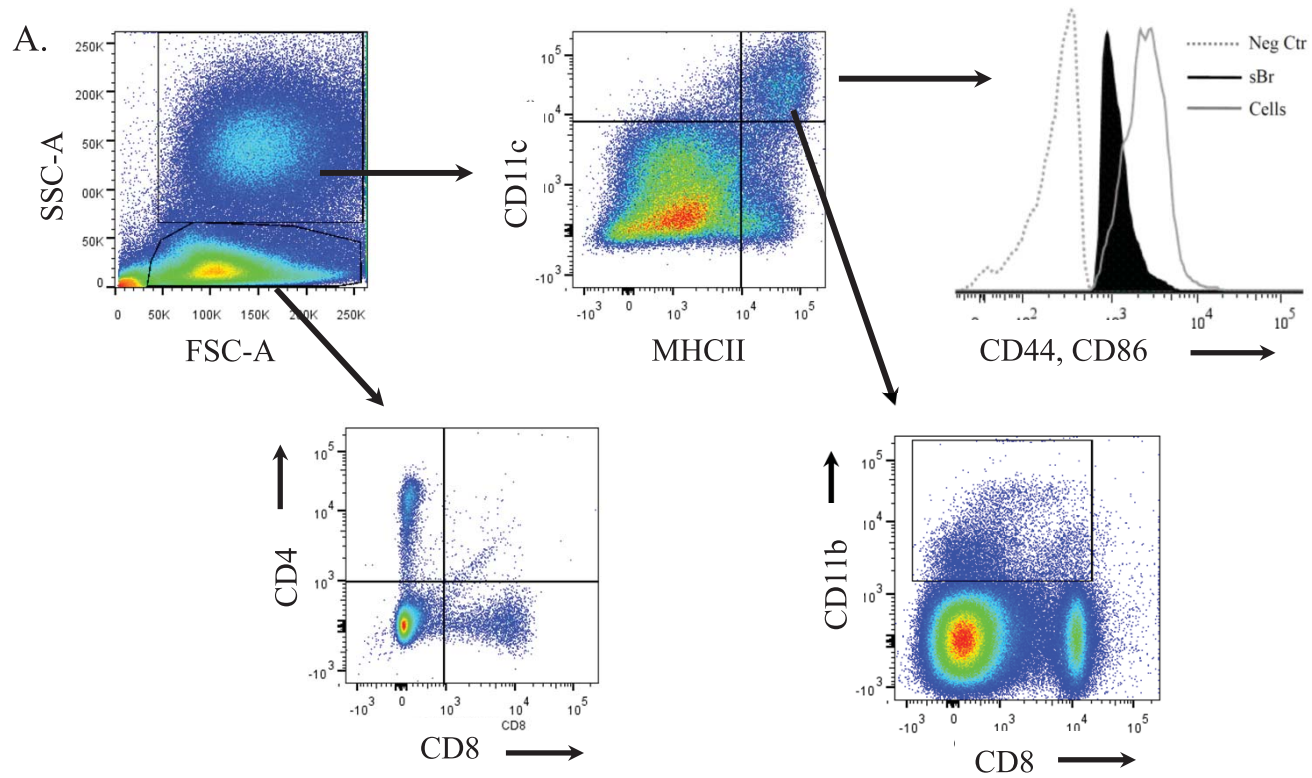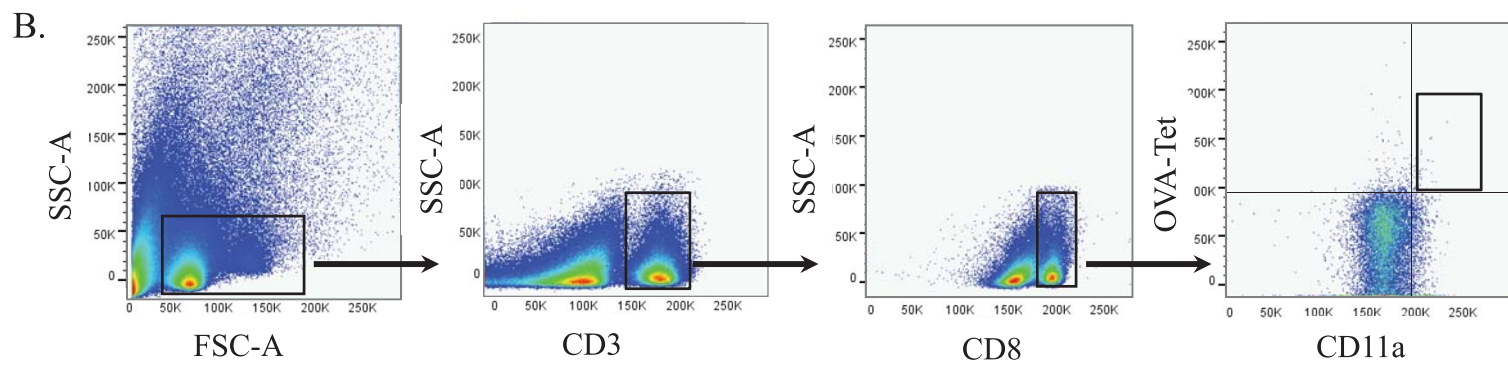

Supplement: Supplementary file 1 — In supplementary Table 1 toxicity parameters (liver function, bronchoalveolar lavage (BAL) protein analysis and total WBCs) determine that sBr treatment was not harmful over the three week treatment course. Supplementary Figure 1 determines that sBr treatment, throughout OVA/alum sensitization, did not alter the BAL cellular differential. In order to determine if the co-localization of i.p. sBr treatment and i.p OVA/alum sensitization resulted in the reduced allergic airway disease (AAD) the immunization and sBr treatment were separated. Supplementary Figure 2 provides data showing that sBr i.p. treatment still reduced BAL leukocytes at AAD, with subcutaneous OVA/alum sensitization. Supplementary Figure 3 depicts the general gating strategies for flow cytometry and Supplementary Figure 4 illustrates the reduction of DC subsets (in the mesenteric lymph nodes) when sBr is administered throughout OVA/Alum sensitization. [file 702196.f1.zip › FigS3.pdf]

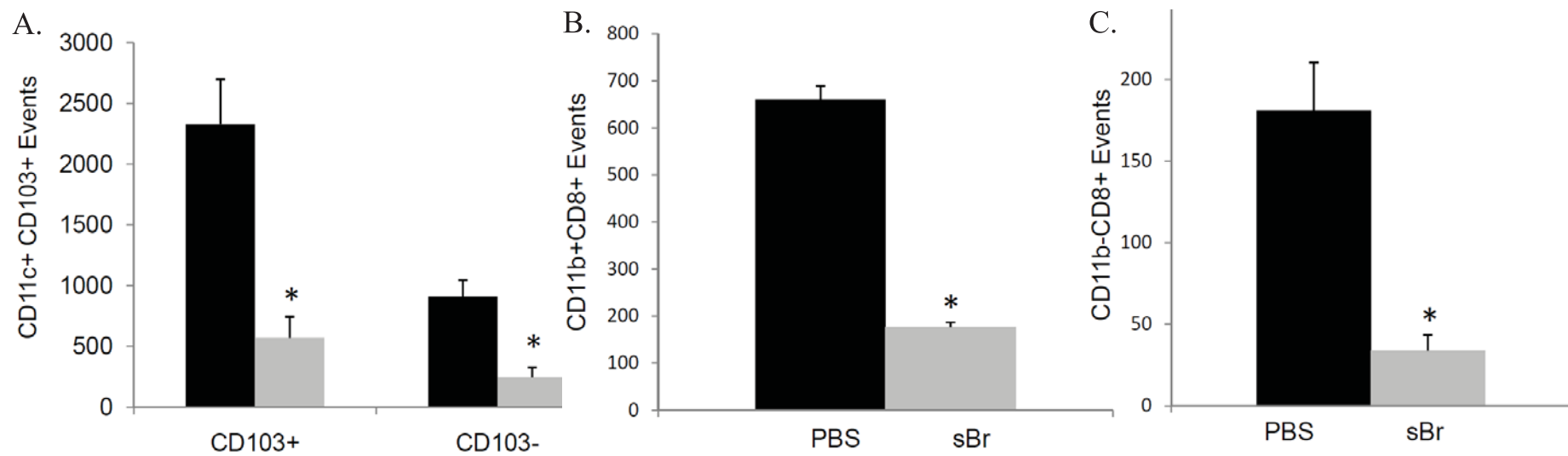

Supplement: Supplementary file 1 — In supplementary Table 1 toxicity parameters (liver function, bronchoalveolar lavage (BAL) protein analysis and total WBCs) determine that sBr treatment was not harmful over the three week treatment course. Supplementary Figure 1 determines that sBr treatment, throughout OVA/alum sensitization, did not alter the BAL cellular differential. In order to determine if the co-localization of i.p. sBr treatment and i.p OVA/alum sensitization resulted in the reduced allergic airway disease (AAD) the immunization and sBr treatment were separated. Supplementary Figure 2 provides data showing that sBr i.p. treatment still reduced BAL leukocytes at AAD, with subcutaneous OVA/alum sensitization. Supplementary Figure 3 depicts the general gating strategies for flow cytometry and Supplementary Figure 4 illustrates the reduction of DC subsets (in the mesenteric lymph nodes) when sBr is administered throughout OVA/Alum sensitization. [file 702196.f1.zip › FigS4.pdf]
